# Prognostic implications of polycomb proteins ezh2, suz12, and eed1 and histone modification by H3K27me3 in sarcoma

**DOI:** 10.1186/s12885-018-4066-6

**Published:** 2018-02-07

**Authors:** Yong Jin Cho, Soo Hee Kim, Eun Kyung Kim, Jung Woo Han, Kyoo-Ho Shin, Hyuk Hu, Kyung Sik Kim, Young Deuk Choi, Sunghoon Kim, Young Han Lee, Jin-Suck Suh, Joong Bae Ahn, Hyun Cheol Chung, Sung Hoon Noh, Sun Young Rha, Sung-Taek Jung, Hyo Song Kim

**Affiliations:** 1 Department of Orthopedic Surgery, Chosun University College of Medicine, Gwangju, South Korea; 2 Anatomic Pathology Reference Lab, Seegene Medical Foundation, Seoul, South Korea; 3 Department of Pathology, Yonsei University College of Medicine, Seoul, South Korea; 4 Department of Pediatric Hemato-Oncology, Yonsei University College of Medicine, Seoul, South Korea; 5 Department of Surgery, Yonsei University College of Medicine, Seoul, South Korea; 6 Department of Urology, Yonsei University College of Medicine, Seoul, South Korea; 7 Department of Obstetrics and Gynecology, Yonsei University College of Medicine, Seoul, South Korea; 8 Department of Radiology, Yonsei University College of Medicine, Seoul, South Korea; 9 Division of Medical Oncology, Department of Internal Medicine, Yonsei University College of Medicine, 134 Shinchondong, Seodaemun-gu, Seoul, 120-752 South Korea; 10 Department of Orthopedic Surgery, Chonnam National University Hospital, Gwangju, South Korea

**Keywords:** Polycomb repressive complex, H3K27me3, Sarcoma

## Abstract

**Background:**

Polycomb repressive complex 2 (PRC2; formed by EZH2, SUZ12, and EED protein subunits) and PRC1 (BMI1 protein) induce gene silencing through histone modification by H3K27me3. In the present study, we characterized the PRC expression pattern and its clinical implication in sarcoma.

**Methods:**

Using immunohistochemistry, we analyzed PRC expression in 105 sarcoma patients with 5 subtypes: synovial sarcoma (*n* = 18), rhabdomyosarcoma (*n* = 28), Ewing sarcoma (*n* = 15), osteosarcoma (*n* = 30), and others (*n* = 14).

**Results:**

The median age at diagnosis in the patient cohort was 26.8 years (range: 1–78 years) and the male-to-female ratio was 1:4. Initial disease presentation was locoregional disease in 83% of patients and initial metastatic disease in the remaining 17%. PRC expression was not significantly different according to histologic subtype (*P* = 0.400). Overall survival (OS) was significantly poor for SUZ12 ^high^ (*P* = 0.001), EED1 ^high^ (*P* = 0.279), and H3K27me3 ^high^ (*P* = 0.009). Ultimately, patients with PRC2^high^ had significantly inferior OS than the no expression group (P = 0.009). In the Cox proportional hazard model adjusted for stage, histologic grade, surgery, margin and initial metastasis, SUZ12 expression (*P* = 0.020, HR 29.069, 95% CI 1.690–500.007), H3K27me3 (*P* = 0.010, HR 3.743, 95% CI 1.370–10.228) expression was significantly associated with shorter OS.

**Conclusion:**

We detected PRC expression in various sarcomas and demonstrated its independent negative prognostic role, suggesting the PRC axis as promising therapeutic target for treating sarcoma.

## Background

Sarcomas are a rare and highly heterogeneous group of neoplasms originating from bone and soft tissue and account for < 1% of all human malignancies [1, 2]. Although surgical resection is the primary treatment for localized disease, more than 40% of cases ultimately experience tumor recurrence, resulting in < 12 months of overall survival (OS) [3]. For patients with these tumors, mortality is high and the local and/or systemic tumor burden results in significant morbidity.

Gene silencing though histone deacetylation, histone methylation, and DNA methylation by deregulated histone deacetylase (HDAC), histone methyltransferase, and DNA methyltransferase has been widely investigated in oncology studies. Among them, polycomb group (PcG) proteins, as epigenetic chromatic modifiers, exhibit histone methyltransferase activity. These proteins regulate various physiologic processes, such as cell cycle, embryogenic development, genetic imprinting, and oncogenesis. The polycomb group protein consists of multiprotein complexes, including polycomb repressive complexes (PRC1) involved in the maintenance of gene silencing and PRC2, which initiates gene silencing. PRC2, containing the enhancer of zeste homologue 2 (EZH2), induces histone H3 trimethylation at lysine 27 (H3K27me3) through histone methyltransferase activity. PRC1, which contains BMI-1 (B lymphoma Mo-MLV insertion region 1 homolog), is recruited to DNA by binding to the H3K27me3 mark and mediates the ubiquitylation of histone 2A at lysine 119. Such coordinated action of PRC2 and PRC1 associated with H3K27me3 on chromatin results in transcriptional repression [4]. The clinical relevance of PRC protein expression has been studied in various solid tumors, such as colorectal cancer, stomach cancer, and lymphoma [5–7]. However, PRC expression and its clinical implications in sarcoma have not been widely investigated.

In this study, we investigated PRC expression in various subtypes of sarcoma and evaluated its clinical relevance. We then analyzed the prognostic potential to provide a practical guide as a diagnostic and therapeutic strategy.

## Methods

### Patients and tissue samples

This study was conducted in a consecutive cohort of patients who were pathologically diagnosed with synovial sarcoma, rhabdomyosarcoma, Ewing sarcoma, osteosarcoma, mesenchymal chondrosarcoma, and epithelioid sarcoma between 1994 and 2013 at the Yonsei Cancer Center. A total of 105 formalin-fixed, paraffin-embedded (FFPE) tissue blocks were available for examination of PRC expression. Fortunately, the samples obtained are all origin site specimens and the adjuvant therapy was not previously performed. All hematoxylin and eosin (H&E) slides were independently reviewed by two experienced pathologists (E.K.K. and S.H.K).

Clinicopathologic variables such as sex, age, tumor histology and grade, tumor location, tumor stage, initial presentation of disease, and status of the resection margin were reviewed retrospectively from electronic medical records. Tumors were graded according to the FNCLCC (French Federation Nationale des Centre de Lutte Contre le Cancer) [8]. Staging was determined using the 7th edition American Joint Committee on Cancer guideline of tumor, node, and metastasis (TNM) classification. The study was approved by the institutional review board of Severance Hospital.

### Immunohistochemical staining and assessment

Tissue microarray blocks were prepared as follows: the hematoxylin-eosin slides were reviewed and representative formalin-fixed, paraffin-embedded (FFPE) archival blocks were selected for each case. Tumors were confirmed and marked on the slides under a microscope. Two or three different representative tumor areas per case were selected and used to prepare the tissue microarrays. Core tissue biopsies (3 mm in diameter) were taken from individual FFPE blocks (donor blocks) and arranged in recipient paraffin blocks (tissue array blocks) using a trephine apparatus. All tissue microarray blocks were confirmed to contain suitable sarcoma lesions with more than 50% of the core area after hematoxylin and eosin staining. The original H&E slides were reviewed by two pathologists (E.K.K and S.H.K).

Immunohistochemistry (IHC) staining for EZH2, SUZ12, EED1, and H3K27me3 was performed on the tissue microarray blocks using a standard protocol with a Ventana automatic immunostainer (Ventana, Benchmark, Tuscan, AZ, USA) [9]. The primary antibodies used in this study were as follows: EZH2 (#18–7395, dilution 1:100, clone ZMD.309; Invitrogen, Carlsbad, CA, USA), SUZ12 (dilution 1:50, clone SUZ220A, Abcam, Cambridge, MA, USA), EED1 (dilution 1:100, clone 163C, Abcam), H3K27me3 (dilution 1:100, clone C36B11; Cell Signaling Technology, Danvers, MA, USA). After deparaffinization, heat-induced antigen retrieval was performed using citrate buffer (CC1 protocol; Ventana) at pH 6.0. Reactivity was detected using the Ultra-View detection kit (Ventana). Positivity for each marker was determined and scored independently by two pathologists (E.K.K and S.H.K.). The percentage and intensity of positive tumor cells were recorded by manually counting representative fields of each case. EZH2, SUZ12, EED1, and H3K27me3 showed nuclear expression. For EZH2, SUZ12, EED1, and H3K27me3, staining intensity (0, none to weak; 1, moderate to strong) and the proportion of positive tumor cell nuclei (0, < 10%; 1, ≥ 10 and < 75%; 2, ≥ 75%) were semi-quantitatively graded as described. Based on the staining intensity multiplied by the proportion of positive nuclei, protein expression was scored as low (0), intermediate (1), or high (2). The cutoff value for high expression was set as a score of 2. [7] High PRC2 expression was defined as cases that were positive for all PRC2 proteins (EZH2, SUZ12, and EED1).

### Statistical methods

SPSS version 18.0 (SPSS, Inc., Chicago, IL, USA) was used for statistical analyses. The correlation between PRC expression and clinicopathologic variables was analyzed using independent sample *t*-test for continuous variables and chi-square test for discrete variables. For survival analysis, OS was defined as the time interval between the diagnosis of metastatic/recurrent disease and death or last follow-up. Survival analysis was performed using the Kaplan-Meier method with the log-rank test. Multivariate analyses for overall survival were performed using the Cox proportional hazards model. The accepted level of statistical significance was *P* < 0.05.

## Result

### Patient characteristics

Baseline clinicopathologic characteristics of the patients are summarized in Table 1. Among the total of 105 cases, 62 were male (59.0%) with a median age at the time of diagnosis of 26.8 years (range 1–78). Histologic subtypes were as follows: osteosarcoma (*n* = 30, 29%), followed by rhabdomyosarcoma (*n* = 28, 27%), synovial sarcoma (*n* = 18, 17%), and Ewing sarcoma (*n* = 15, 14%). Most cases were grade 3 (*n* = 99, 95%) and more than one-third were located in the lower extremities (*n* = 44, 42%). Most patients had no distant metastasis at the time of diagnosis (83%) and underwent surgical resection (n = 99, 95%). Adjuvant chemotherapy was given in 63 patients (65%), and 38 of these (40%) received concurrent radiotherapy.

**Table 1:** Baseline characteristics

Variables			All PRC2 expression		*P* value
		*N* (%)	Low or Intermediate	High	
Sex	Male	62 (59%)	59 (56%)	3 (3%)	0.965
	Female	43 (41%)	41 (39%)	2 (2%)	
Age at diagnosis (year, median, range)		26.8 (1–78)	26.3 ± 18.7	35.6 ± 19.7	0.280
Diagnosis	Synovial sarcoma	18 (17%)	18 (17%)	0 (0%)	0.400
	Rhabdomyosarcoma	28 (27%)	25 (24%)	3 (3%)	
	Ewing sarcoma	15 (14%)	14 (13%)	1 (1%)	
	Osteosarcoma	30 (29%)	29 (28%)	1 (1%)	
	Others	14 (13%)	14 (13%)	0 (0%)	
Primary Site	Head/Neck	20 (19%)	20 (19%)	0 (0%)	0.512
	Trunk	29 (28%)	27 (26%)	2 (2%)	
	Upper Extremity	12 (11%)	12 (11%)	0 (0%)	
	Lower Extremity	44 (42%)	41 (39%)	3 (3%)	
Histologic Grade	Grade 1	4 (4%)	4 (4%)	0 (0%)	0.853
	Grade 2	2 (2%)	2 (2%)	0 (0%)	
	Grade 3	99 (95%)	94 (90%)	5 (5%)	
Initial distant metastasis	No	83 (83%)	80 (80%)	3 (3%)	0.531
	Yes	17 (17%)	16 (16%)	1 (1%)	
Surgery	No	6 (6%)	5 (5%)	1 (1%)	0.259
	Yes	99 (95%)	95 (91%)	4 (4%)	
Resection margin	R0	68 (69%)	66 (67%)	2 (2%)	0.636
	R1	29 (29%)	27 (27%)	2 (2%)	
	R2	2 (2%)	2 (2%)	0 (0%)	
Stage	I or II	53 (50%)	52 (49%)	1 (1%)	0.205
	III or IV	52 (50%)	48 (46%)	4 (4%)	
Chemotherapy	No	33 (35%)	30 (31%)	3 (4%)	0.081
	Yes	63 (65%)	62 (64%)	1 (1%)	
Radiotherapy	No	57 (60%)	56 (59%)	1 (1%)	0.144
	Yes	38 (40%)	35 (37%)	3 (3%)	

### PRC expression status and clinicopathologic features

**Fig. 1:**
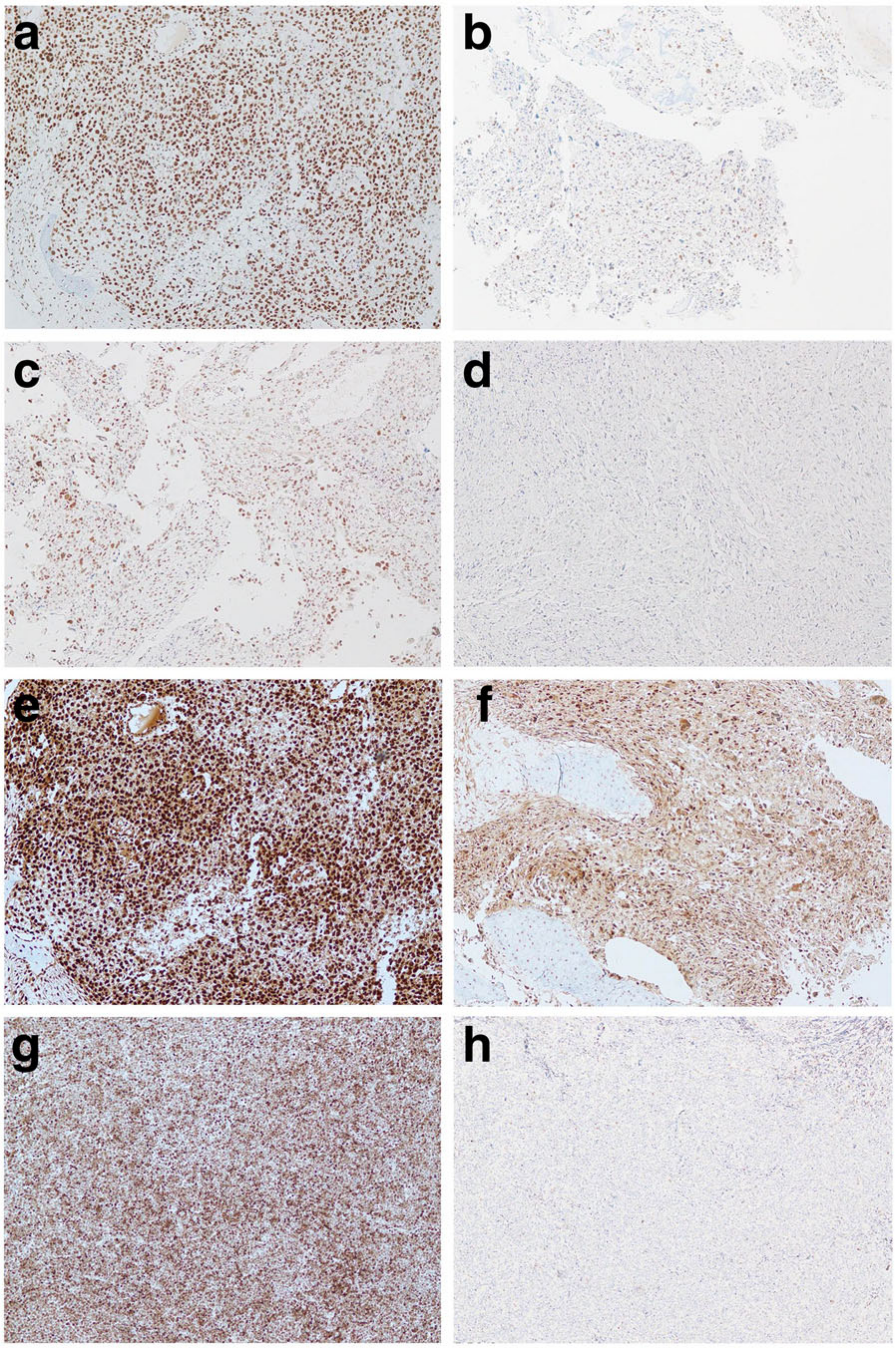
Representative expression of polycomb proteins in sarcoma (×100). **a** High-grade expression of EZH2. **b** Low-grade expression of EZH2. **c**, High-grade expression of SUZ12. **d**, Low-grade expression of SUZ12. **e** High-grade expression of EED1. **f** Low-grade expression of EED1. **g** High-grade expression of H3K27me3. **h**, Low-grade expression of H3K27me3

**Fig. 2:**
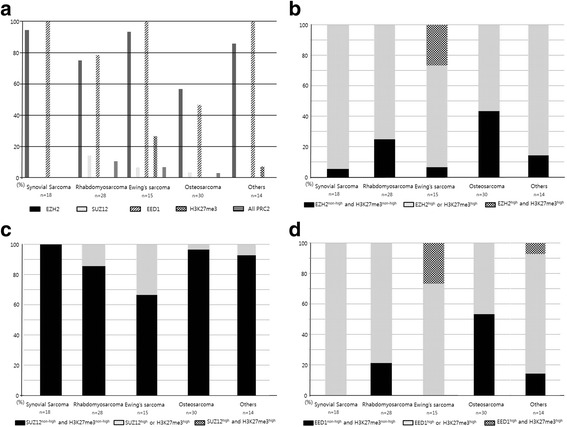
High expression rate of polycomb proteins and H3K27me3 according to subtype. **a** Activation rate for polycomb proteins and H3K27me3. **b** Activation rate of EZH2-H3K27me3. **c** Activation rate of SUZ12-H3K27me3. **d** Activation rate of EED1-H3K27me3

For PRC2 (EZH2, SUZ12, and EED1 protein subunits) and H3K27me3, representative images of PRC-positive and PRC-negative cases are shown in Fig. 1. In Fig. 1. a-b, c-d, e-f and g-h are combinations of samples showing the high and low expression of the same polycomb proteins in the same pathologies. In detail, the pleomorphic tumor cells produce osteoid describing irregular trabeculae in Fig. 1a. The specimen obtained from the patient diagnosed with osteosarcoma has high grade expression of EZH2 staining. Fig. 1b. shows the specimen obtained from patients diagnosed with osteosarcoma with low grade expression of EZH2. c-d, e-f and g-h were diagnosed as osteosarcoma but showed high grade and low grade expression in SUZ12, EED1 and H3K27me3 expression, respectively. The high expression rates of EZH2, SUZ12, and EED1 were 77.1% (*n* = 81), 5.7% (*n* = 6), and 77.1% (n = 81), respectively. Each PRC expression level was significantly different according to the histologic subtype of sarcoma (Fig. 2). As seen in Fig. 2a, SUZ12 and H3K27me3 are relatively low. Fig. 2b. shows that EZH2 and H3K27me3 are low at the same time, one of them is high or both are high, and EZH2 ^high^ or H3K27me3 ^high^ occupies a high ratio as shown by the relatively low H3K27me3. As shown in Fig. 2c. SUZ12 and H3K27me3 are low at the same time, one of them is high, and both of them are high, and SUZ12 ^non-high^ and H3K27me ^non-high^ occupies a high ratio. Finally, Fig. 2d shows that EED1 and H3K27me3 are low at the same time, one of them is high and both of them are high. For the same reason, EED1 ^high^ or H3K27me3 ^high^ seems to occupy a high percentage.The expression patterns of EZH2, SUZ12, EED1, and H3K27me3 were most distinctive; EZH2^high^ and EED1^high^ were frequently observed in all subtypes. SUZ12^high^ and H3K27me3^high^ were uniformly infrequent across all subtypes, whereas H3K27me3^high^ was frequent in Ewing’s sarcoma. There was no significant difference in the PRC2 expression of each protein with sex (*P* = 0.965), primary site of diagnosis (*P* = 0.512), histologic grade (*P* = 0.853), stage at diagnosis (*P* = 0.205), initial distant metastasis (*P* = 0.531), surgery (*P* = 0.259), resection margin (*P* = 0.636), and adjuvant treatment (chemotherapy; *P* = 0.081, radiotherapy; *P* = 0.144). The combined expression of all PRC2 proteins was not significant for clinicopathologic features.

### Survival outcome according to PRC expression

**Fig. 3:**
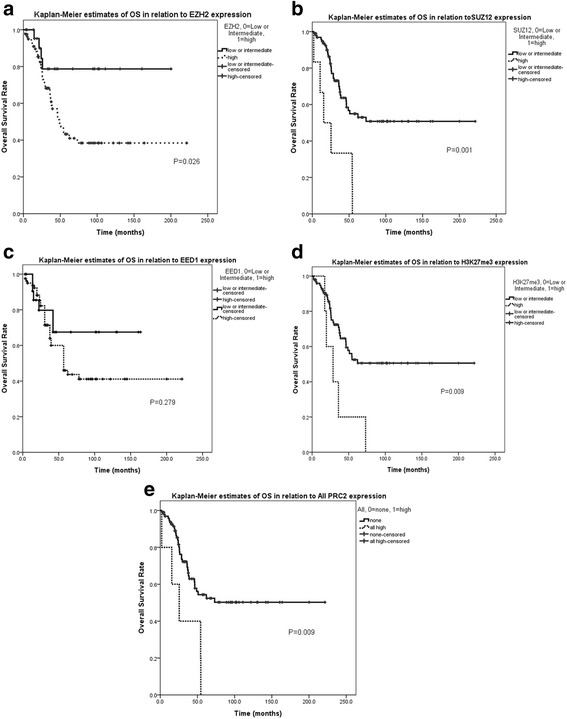
Polycomb proteins affect overall survival of sarcoma patients based on Kaplan-Meier plots (*n* = 105). **a** Overall survival rate according to EZH2 expression in overall sarcoma. **b** Overall survival rate according to SUZ12 expression in overall sarcoma. **c** Overall survival rate according to EED1 expression in overall sarcoma. **d** Overall survival rate according to H3K27me3 expression in overall sarcoma. **e** Overall survival rate according to all PRC2 expression in overall sarcoma

After a median follow-up duration of 33.8 months, 41 patients (39.0%) had died at the time of survival analysis. The 5-year OS for all patients was 48.7% and the 5-year OS rates of the subgroups were as follows: 67.4% for rhabdomyosarcoma, 64.7% for synovial sarcoma, 18.7% for Ewing sarcoma, 52.1% for osteosarcoma, and 22.0% for others (mesenchymal chondrosarcoma or epithelioid sarcoma). In subtype analysis, EZH2^high^ expression was associated with significantly shortened OS (5-year OS rate, 78% vs. 41%, *P* = 0.026, Fig. 3a). The OS was also significantly poor for SUZ12 ^high^ (5-year OS rate, 53% vs. 0%, *P* = 0.001, Fig. 3b), EED1^high^ (5-year OS rate, 67% vs. 44%, *P* = 0.279, Fig. 3c), and H3K27me3^high^ (5-year OS rate, 51% vs. 0%, *P* = 0.009, Fig. 3d). Ultimately, overexpression of PRC2^high^ showed significantly inferior OS compared to the no expression group (5-year OS rate, 52% vs. 0%, *P* = 0.009, Fig. 3e). The Cox proportional hazard model was adjusted for stage, histologic grade, initial metastasis, surgery, margin status, and EZH2, SUZ12, H3K27me3, and all PRC2. Initial metastasis (*P* = 0.013, HR 3.365, 95% CI 1.295–8.745), SUZ12 expression (*P* = 0.020, HR 29.069, 95% CI 1.690–500.007), and H3K27me3 (*P* = 0.010, HR 3.743, 95% CI 1.370–10.228) expression were significantly associated with a shorter OS (Table 2).

**Table 2:** Univariate and multivariate analysis for overall survival

Variables	Univariate		Multivariate	
	5-yr OS	*P*-value	HR (95% CI)	*P*-value
Sex (Male vs. Female)	49% vs. 48%	0.970		
Age (≥20 vs. < 20)	41% vs. 53%	0.690		
Stage(I or II vs. III or IV)	60% vs. 36%	0.020		
Histologic Grade (Grade 1 or 2 vs. Grade 3)	100% vs. 45%	0.043		
Tumor location (Axial vs. Extremity)	42% vs. 52%	0.389		
Initial metastasis (Yes vs. No)	16% vs. 55%	0.001	3.365 (1.295–8.745)	0.013
Surgery (Yes vs. No)	50% vs. 0%	0.045		
Margin status (R0 vs. R1 or R2)	59% vs. 32%	0.036		
EZH2 (non-high vs. high)	78% vs. 41%	0.026		
SUZ12 (non-high vs. high)	53% vs. 0%	0.001	29.069 (1.690–500.007)	0.020
EED1 (non-high vs. high)	67% vs. 44%	0.279		
H3K27me3 (non-high vs. high)	51% vs. 0%	0.009	3.743 (1.370–10.228)	0.010
All PRC2 (non-high vs. high)	52% vs. 0%	0.009		

*HR* hazard ratio, *CI*, confidence interval

## Discussion

We examined the frequency and prognostic impact of PRC expression in various sarcomas. This is the first study to evaluate the prognostic impact of PRC expression in sarcoma subgroups. We found that PRC2 proteins and H3K27me3 were frequently expressed and that overexpression was as an independent prognostic factor for OS.

Accumulating evidence has suggested the controversial values of PRC expression in solid tumors. Wang et al. reported that high EZH2 expression was associated with poor prognosis in colorectal cancer [10], whereas EZH2 expression was associated with relapse-free survival in another study [11]. Similarly to PRC2, BMI1 was also associated with good prognosis in breast cancer and poor prognosis in colorectal cancer [12, 13]. Clinicopathologic heterogeneity including primary tumor, pathologic stage, and chemotherapy or radiotherapy may have contributed to these controversial results. Furthermore, conclusions based on one protein marker may lead to inconsistent results.

In addition to individual markers, combinations of PRC proteins have been reported by several groups. Co-expression of EZH2 and BMI1 was found to be a poor prognostic factor. Coactivation of PRC2 proteins (EZH2, SUZ12, and EED) were reported to be associated with inferior OS in NK T cell lymphoma [14]. Esophageal squamous cell carcinoma, which exhibits high expression of BMI1 and EZH2, showed poor OS and disease-free survival. In our study of sarcoma, all PRC proteins were consistently poor prognostic factors. To obtain additional prognostic information regarding epigenetic pathways, we performed multivariate analyses using combined expression of PRC2 proteins and the association with histone modification H3K27me3. By simultaneously examining the protein complex, we clarified the prognostic value of PRC expression in sarcoma patients.

Synovial sarcoma has been widely studied to determine its association with sarcoma-specific genetic aberration. Synovial sarcoma is defined by a characteristic translocation t(X;18)(p11.2;q11.2), which is observed in > 95% of cases and results in the fusion of SS18 to the SSX1, SSX2, or SSX4 genes [15]. Thus, SS18-SSX fusion leads to disease development by disrupting the epigenetic regulation of gene expression. Soulez et al. demonstrated that the polycomb group proteins RNG1 or BMI are co-localized with SSX focal staining in synovial sarcoma [16]. In addition, poorly differentiated synovial sarcoma showed high expression of EZH2, which was predictive for distant metastasis [17]. Recently, the SS18-SSX fusion protein was reported to be associated with the recruitment of PRC, suggesting polycomb-mediated epigenetic gene regression as a mechanism of oncogenesis in synovial sarcoma cell lines [18].

In a study by Schaefer et al. loss of H3K27 tri-methylation was shown in 51% (51/100) of malignant peripheral nerve sheath tumors and in another study by Prieto-Granada et al., loss of H3K27 tri-methylation was found in 69% (47/68) of malignant peripheral nerve sheath tumors [19, 20]. Arjen et al. reports that malignant peripheral nerve sheath tumors with loss of H3K27 tri-methylation showed inferior survival compared to malignant peripheral nerve sheath tumors with intact H3K27 tri-methylation [21]. Schaefer et al. demonstrated increased loss of H3K27 with increasing histological grade [19]. Combinational effect of genetic aberration, heterogeneity of primary tumor and treatment modality, the role of genetic aberration in the progression of sarcoma can be considered as reasons for these paradoxical findings in sarcoma.

Anthracyclines and ifosfamide, either alone or in combination, are the gold standard treatments for advanced sarcoma [3]. After the failure of conventional first-line cytotoxic chemotherapy, available treatment options such as gemcitabine, trabectedin, and pazopanib have been assessed for the subsequent treatment of sarcoma. However, these treatments are severely limited because of their high toxicities and modest survival benefits; thus, the availability of less toxic agents with novel mechanisms that can improve progression-free survival or OS are crucial. Currently, most studies have focused on the prognostic role of PRC, while the therapeutic role of PRC has not been widely examined. The initiation of gene silencing by polycomb activity has been shown to involve HDAC [22]. The HDAC inhibitor FK228 shows growth inhibitory effects in synovial sarcoma cell lines and in vivo models [23]. Introduction of SYT-SSX cDNA enhanced the sensitivity to FK228, and the growth of synovial sarcoma tumor graft was markedly inhibited by FK228 treatment. Puppe et al. reported that BRCA-1-deficient breast cancer, which overexpress EZH2, was 20-fold more effective compared to BRCA-proficient breast cancer cell lines when the H3K27me3 selective inhibitor DZNep was used [24]. The *S*-adenosyl-methionine (SAM)-competitive inhibitors of EZH2, including tazemetostat (EPZ-6438, E7438) [25], (*R*)-*N*-((4-Methoxy-6-methyl-2-oxo-1,2-dihydropyridin-3-yl)methyl)-2-methyl-1-(1-(1-(2,2,2-trifluoroethyl)piperidin-4-yl)ethyl)-^1^*H*–indole-3-carboxamide (CPI-1205) [26], GSK2816126, are effective and selective small molecular against EZH2. Tazemetostat, CPI-1205, and GSK2816126, are currently performed in the clinical trials in different cancer types, including lymphomas, kidney tumors, synovial sarcoma, epitheliod sarcoma, mesothelioma, advanced solid tumors, and ovarian cancer. Therefore, PRC are putative therapeutic targets in sarcoma.

The main limitations of our study include its retrospective nature and patient selection. Therefore, our findings should be validated in an independent sarcoma cohort and the response data to HDAC inhibitors should be examined in future clinical trials. Higher magnification of IHC staining and normal tissue comparing may have been better understood, but in this study it is an important limitation that we could not prepare it beforehand.

## Conclusion

We determined PRC expression in various sarcomas and demonstrated the independent negative prognostic role of these proteins. Our results suggest the PRC axis as a promising therapeutic target for the treatment of sarcoma.
